# Supplementary material for: Influence of Age and Immunostimulation on the Level of Toll-Like Receptor Gene (TLR3, 4, and 7) Expression in Foals
Source: Animals (Basel). 2020 Oct 26;10(11):1966. doi: 10.3390/ani10111966 (PMC7692595; doi:10.3390/ani10111966)
Supplement: Supplementary file 1 [file animals-10-01966-s001.zip › animals-920942-supplementary/animals-920942 Supplementary Tables S1,S2, Figure S1.docx]

Article

Supplementary Materials: Influence of Age and Immunostimulation on the Level of Toll-Like Receptor Gene (*TLR3, 4,* and *7*) Expression in Foals

Anna Migdał ^1,^*, ŁukaszMigdał^1^, Maria Oczkowicz ^2^, Adam Okólski ^3^ and
Anna Chełmońska-Soyta^4,5^

**Table S1.** Mastermix reaction.

| **Reagent** | **Volume (µL)** |
| --- | --- |
| GoTaq®Probe qPCR Master | 5 |
| cDNA | 1.5 |
| TaqMan | 1 |
| H_2_O | 2.5 |

**Table S2.** Reaction efficiency.

| **Gen** | **Full Name**  **of the Gene** | **Dye** | **Amplicon Length (bp)** | **Reaction Efficiency %** |  |
| --- | --- | --- | --- | --- | --- |
| *TLR3* | Toll-Like receptor 3 | FAM | 125 | 90.25 | |
| *TLR4* | Toll-Like receptor 4 | FAM | 91 | 84 |  |
| *TLR7* | Toll-Like receptor 7 | VIC | 64 | 86.5 |  |
| *SDHA* | succinate dehydrogenase complex subunit A | VIC | 56 | 88.5 |  |
| *HPRT* | Hypoxantinephsophoribosyl transferase | VIC | 84 | 93 | |

| 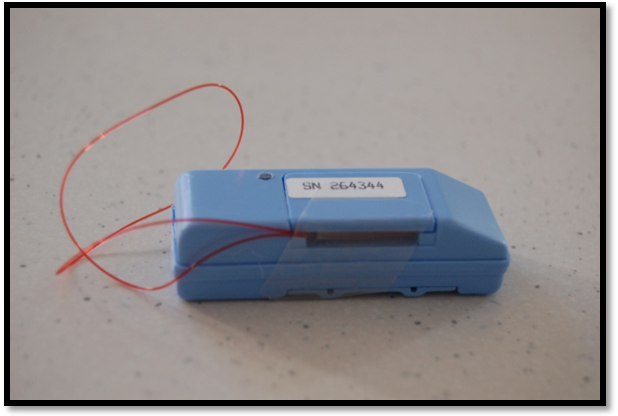  **A** | 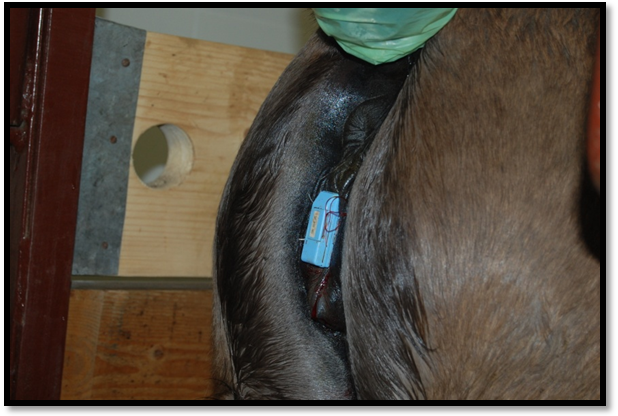  **B** |
| --- | --- |

**Figure S1.** Birth system alarm. **A**—Signal transmitter; **B**—Transmitter sewn in mare’s vulva
