# Supplementary material for: Influence of Age and Immunostimulation on the Level of Toll-Like Receptor Gene (TLR3, 4, and 7) Expression in Foals
Source: Animals (Basel). 2020 Oct 26;10(11):1966. doi: 10.3390/ani10111966 (PMC7692595; doi:10.3390/ani10111966)
Supplement: Supplementary file 1 [file animals-10-01966-s001.zip › animals-920942-supplementary/animals-920942-Supplementary File 1.doc]

Article

Supplementary Materials: Influence of Age and Immunostimulation on the Level of Toll-Like Receptor Gene (*TLR3, 4,* and *7*) Expression in Foals

Anna Migdał ^1,^*, Łukasz Migdał ^1^, Maria Oczkowicz ^2^, Adam Okólski ^3^ and
Anna Chełmońska-Soyta ^4,5^

RNA Isolation and Reverse Transcription

RNA was isolated from blood using TEMPUS SPIN (Ambion®). Isolated total RNA were quantified using Nanodrop 2000 (ThermoScientific, Wilmington, DE, USA) and quality were evaluated on 2% agarose gel with addition of ethidium bromide. 1 µg of total RNA were transcribed on cDNA using High Capacity cDNA Reverse Transcription Kit (Applied Biosystems) according to protocol. For random RNA samples “No-RT” were performed.

Real-Time PCR

Gene expression were performer on Illumina Eco (Illumina) using TaqMan®MGB with fluorescent probes labelled with FAM and VIC. For each sample reaction was performer in triple replication in 10 µL volume. Mastermix were prepared according to protocol and contained: Master Mix GoTaq®Probe qPCR, cDNA, molecular probes and 20× concentrated TaqMan gene expression assay. Ultrapure water were addend to volume of 10 µL (Table S1). Reaction conditions were set according to GoTaq®Probe qPCR Master Mix protocol: polimerase activation (2 min in 95 ^°^C) and 40 cycles −95 ^°^C by 15 s (denaturation) and 60 ^°^C by 1 min (annealing/extension).

Using molecular probes with different fluorescent label (FAM and VIC), we performer multiplex reactions for every analyzed gene in presence of two housekeeping genes (SDHA and HPRT): *TLR4 and SDHA, TLR7 and HPRT (first reaction), TLR3 and HPRT (second reaction).* Reaction efficiency for every gene were estimated using standard curve (Table S2). Efficiency (E) were calculated according to equation E = 10^[−1/slope]^_,_ from slope of obtained regression line. Relative number of transcripts were calculated according to equation 1/E^(Ct)^, [E = efficiency (10^[−1/slope]^), Ct (threshold cycle)]

Blood Morphotic Parameters Analysis

1 mL of blood were collected into EDTK3 tubes for hematology analysis which were performed immediately after blood collection.

Erythrocyte counts were performed in 200-fold dilution of saline solutions using the Thoma chamber, leukocyte counts in 20-fold dilution of Türk’s solutions using the Bürker chamber. The hematocrit was analysed by the microhematocrit method by capillary centrifugation (2 min, 8000 rpm).

To perform a leukocytogram we prepared two blood smears, which were stained according to May Grünwald-Giemza methods (Mariańska 2006), and were analyzed using a stereoscopic microscope (SMZ 1000, Nikon, Japan over 1000×). In smear we counted 200 leukocytes, which were later divided into populations of lymphocytes, monocytes, neutrophils, basophils, intensive acid granulocytes (eosinophils). Comparing the percentages with the total leukocyte count were used to calculate absolute value of the various blood cells.

Total hemoglobin was estimated by Drabkin’s cyanmethemoglobin method with a spectrometer SmartSpecPlus (Bio-Rad).
